# Supplementary material for: Biomarkers to Predict Lethal Radiation Injury to the Rat Lung
Source: Int J Mol Sci. 2023 Mar 15;24(6):5627. doi: 10.3390/ijms24065627 (PMC10053311; doi:10.3390/ijms24065627)
Supplement: Supplementary file 1 [file ijms-24-05627-s001.zip › ijms-2112153-supplementary.pdf]

Supplemental Figure S1

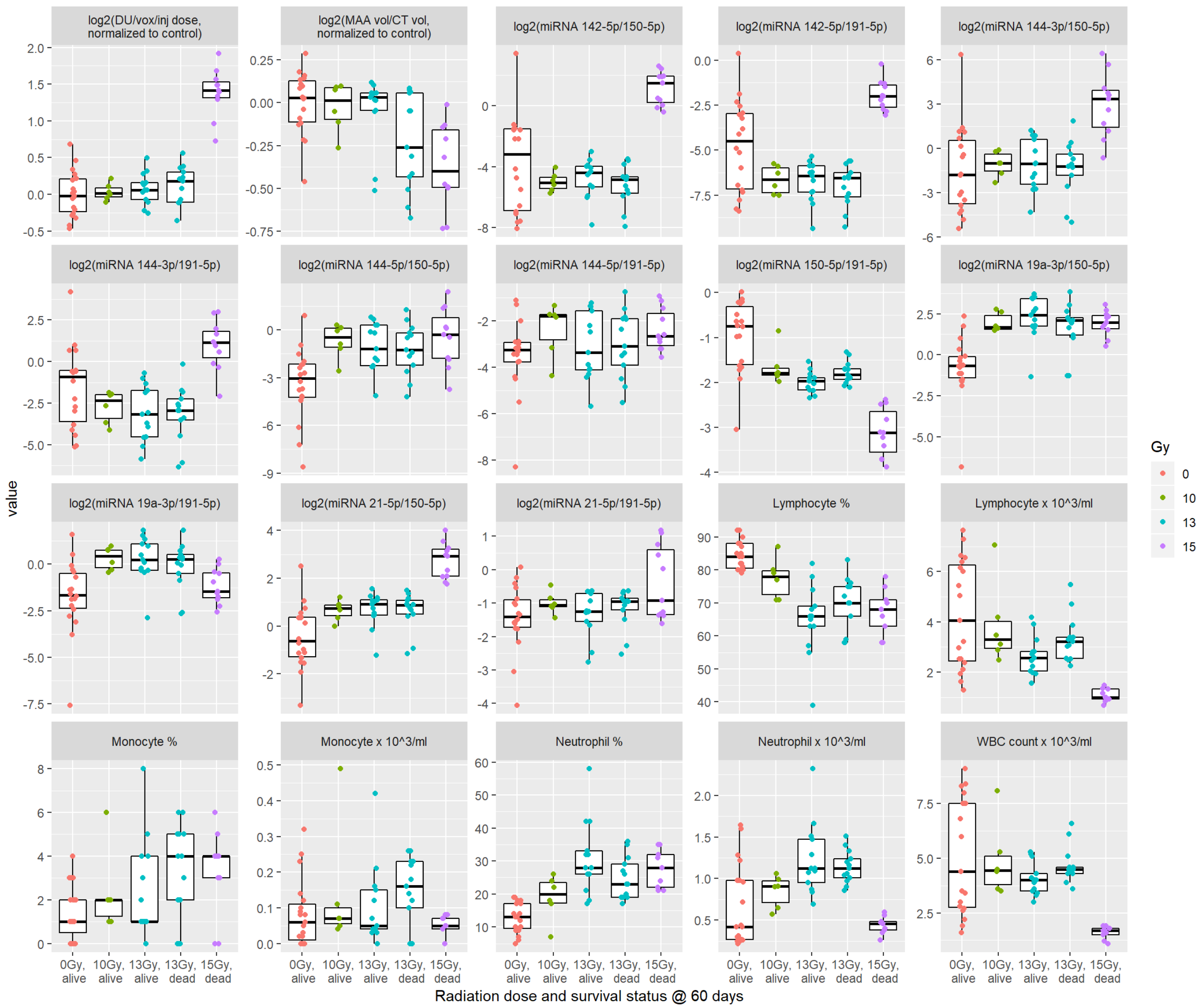

Supplemental Figure S1. Complete data set plotted by outcome.

Supplemental Figure S2

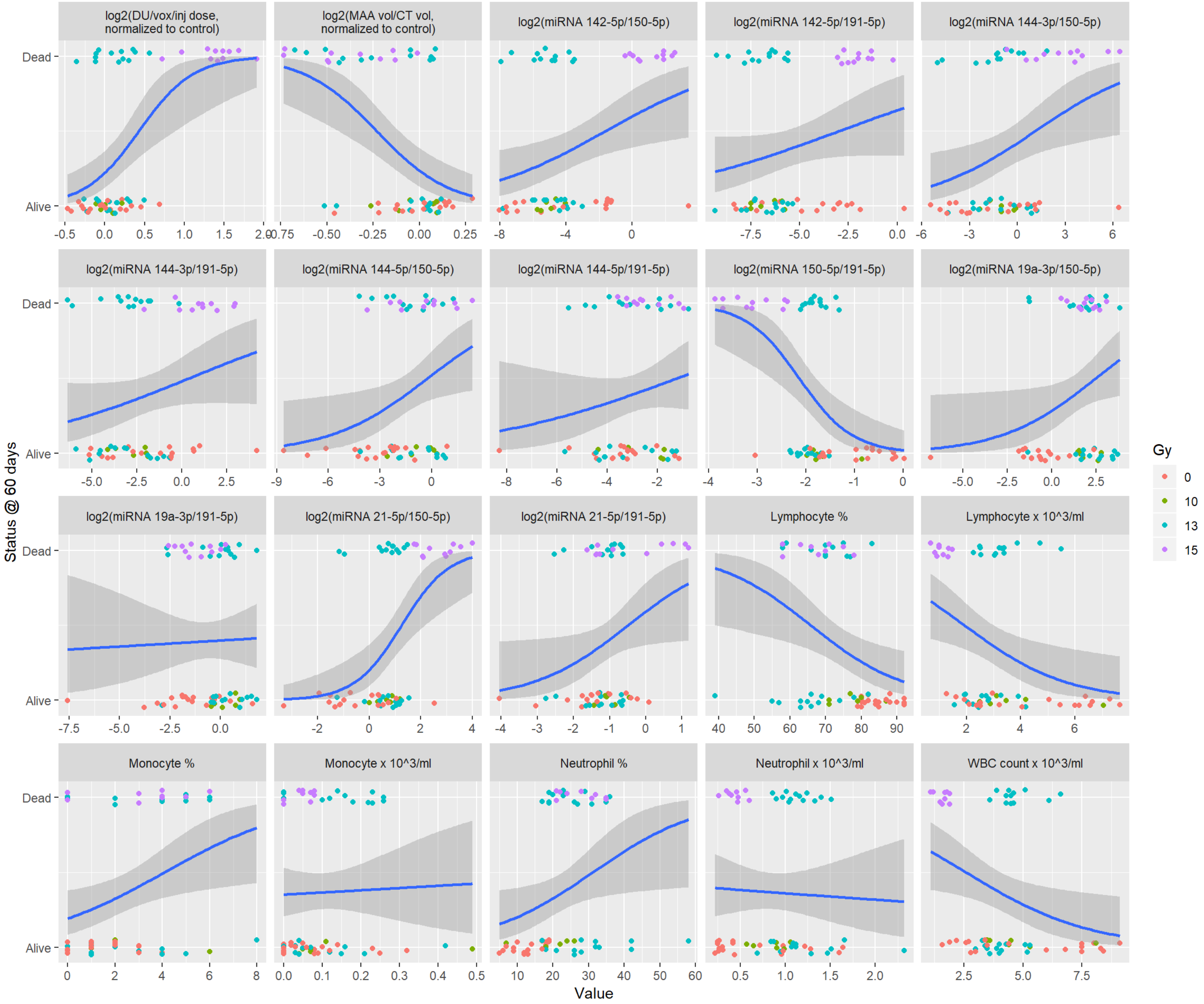

Supplemental Figure S2. Visualization of the complete data set by univariate strip plots of predictors of 60-day survival with overlaid logistic regression curves.
